# Nicotinamide Riboside Improves Stemness of Human Adipose-Derived Stem Cells and Inhibits Terminal Adipocyte Differentiation

**DOI:** 10.3390/ph16081134

**Published:** 2023-08-10

**Authors:** Somaiah Chinnapaka, Hamid Malekzadeh, Zayaan Tirmizi, José A. Arellano, Asim Ejaz

**Affiliations:** Department of Plastic Surgery, University of Pittsburgh, Pittsburgh, PA 15261, USA

**Keywords:** adipose stem cells, adipose tissue, stemness, differentiation, proliferation, nicotinamide riboside, reactive oxygen species

## Abstract

Adipose tissue plays a crucial role in maintaining metabolic homeostasis by serving as a storage site for excess fat and protecting other organs from the detrimental effects of lipotoxicity. However, the aging process is accompanied by a redistribution of fat, characterized by a decrease in insulin-sensitive subcutaneous adipose depot and an increase in insulin-resistant visceral adipose depot. This age-related alteration in adipose tissue distribution has implications for metabolic health. Adipose-derived stem cells (ASCs) play a vital role in the regeneration of adipose tissue. However, aging negatively impacts the stemness and regenerative potential of ASCs. The accumulation of oxidative stress and mitochondrial dysfunction-associated cellular damage contributes to the decline in stemness observed in aged ASCs. Nicotinamide adenine dinucleotide (NAD+) is a crucial metabolite that is involved in maintaining cellular homeostasis and stemness. The dysregulation of NAD+ levels with age has been associated with metabolic disorders and the loss of stemness. In this study, we aimed to investigate the effects of nicotinamide riboside (NR), a precursor of NAD+, on the stemness of human ASCs in cell culture. Our findings reveal that adipogenesis is accompanied by an increase in mitochondrial activity and the production of reactive oxygen species (ROS). However, treatment with NR leads to a reduction in mitochondrial activity and ROS production in ASCs. Furthermore, NR administration improves the stemness-related genes expression in ASCs and mitigates their propensity for adipocyte differentiation. These results suggest that NR treatment holds promise as a potential strategy to rejuvenate the stemness of aged ASCs. Further investigations, including in vivo evaluations using animal models and human studies, will be necessary to validate these findings and establish the clinical potential of this well-established drug for enhancing the stemness of aged stem cells.

## 1. Introduction

Adipose tissue serves a crucial role in maintaining overall metabolic balance, insulin sensitivity, and immune modulation. It acts as a storage site for excess fatty acids, preventing their accumulation in other organs and the associated negative consequences, such as steatosis and lipotoxicity [1]. Ageing is associated with the progressive deterioration of cellular function and is one of the greatest risk factors for developing a large cohort of debilitating diseases [2]. Age-associated insulin resistance, a prevalent metabolic disorder, affects a significant portion of the elderly population in the United States, leading to increased mortality rates, reduced functional status, higher hospitalization risk, and a substantial burden on healthcare systems [3]. Although the connection between obesity and diabetes is well-established, the relationship between aging and type 2 diabetes remains less understood [4] In fact, the correlation between declining glucose tolerance and aging was first observed nearly a century ago in 1921 [5]. Since then, numerous studies have identified reduced insulin sensitivity as the primary contributor to age-related impairments in glucose metabolism [6,7].

Adipose tissue can be categorized into two main depots based on its anatomical localization: subcutaneous and visceral depots. The insulin sensitivity of these depots differs, with subcutaneous adipose tissue displaying higher sensitivity to insulin compared to the more insulin-resistant visceral depot [8]. The gradual loss of subcutaneous adipose tissue volume associated with aging leads to diminished glucose and lipid uptake, resulting in a phenomenon known as “lipid overflow” and the ectopic deposition of lipids in muscles and liver. This contributes to the development of insulin resistance [9].

Comprising various cell types, adipose tissue can be enzymatically digested to yield two primary fractions: the adipocyte fraction and the stromal vascular fraction (SVF) [10]. The SVF consists of adipose-derived stem cells (ASCs), lymphocytes, endothelial cells, pericytes, and fibroblasts [11]. ASCs are characterized by their immune-phenotypic profile as CD34 + CD90 + CD29 + CD45-CD31- cells of mesenchymal origin, possessing the ability to differentiate into adipocytes, as well as bone and cartilage cells [12,13]. In white adipose tissue, these cells predominantly reside in the vascular stroma surrounding small blood vessels, where they can undergo proliferation and adipogenic differentiation [12]. At the transcriptional level, the primary regulators of adipogenesis are the key transcription factors peroxisome proliferator-activated receptor γ (PPARγ), CCAAT/enhancer binding protein α (C/EBPα) and CCAAT/enhancer binding protein β [14]. These transcription factors play essential roles in inducing growth arrest and orchestrating the sequential expression of genes involved in lipid metabolism. Notably, they regulate the expression of key adipocyte-specific hormones such as adiponectin and leptin. ASCs play a crucial role in adipose tissue regeneration through their capacity for proliferation, self-renewal, and differentiation. However, aging is associated with a decline in the stemness properties of ASCs [15,16]. The age-related reduction in subcutaneous fat depot size is thought to be influenced by alterations in the replication and differentiation capabilities of ASCs [17,18,19]. Accumulation of oxidative stress within adipose tissue during aging has been proposed as a contributing factor that negatively affects the differentiation capacity of ASCs [17]. Nevertheless, the exact mechanisms underlying the loss of stemness in ASCs during aging remain unclear. Recent studies have highlighted a connection between decreased autophagy, increased mechanistic targets of rapamycin (mTOR) pathway activity, and the accumulation of reactive oxygen species (ROS) with the loss of stemness and premature senescence induction in ASCs [20,21,22]. Understanding the mechanisms underlying the age-related decline in ASC stemness is crucial for developing strategies to mitigate its impact. Furthermore, investigating the potential therapeutic interventions that can preserve or restore the stemness properties of ASCs may hold promise for combating age-related adipose tissue dysfunction and its associated metabolic disorders.

Nicotinamide adenine dinucleotide (NAD) and its related co-enzymes, including NADH, NADP^+^, and NADPH, play pivotal roles as central mediators in various metabolic processes. NAD itself is indispensable for maintaining metabolic homeostasis, as it acts as a receptor for hydride groups, accepting electrons from other metabolites in diverse metabolic pathways to form NADH. The transfer of electrons facilitated by NAD/NADH is crucial for fundamental cellular functions such as DNA synthesis, mitochondrial respiration, energy production, and overall metabolism [23]. NAD levels tend to decline with age in multiple tissues and organs. The disruption of NAD metabolites can occur due to different metabolic stressors, including overnutrition, type 2 diabetes, circadian disruption, DNA damage, postpartum conditions, heart failure, and tissue injuries [23,24,25]. In light of this, NAD precursors have recently emerged as promising therapeutic agents for age-related regenerative decline and associated diseases. The administration of NAD precursors, nicotinamide mononucleotide (NMN), which decreases with age in mammals, effectively restores NAD levels in aged mice tissues and successfully reverses age-related stem cell dysfunction in various organ systems, including skeletal muscle, brain, skin, and endothelium. However, clinical use of NMN is challenged by the dose-dependent adverse hepatotoxicity [26]. Notably, the NAD precursor nicotinamide riboside (NR) has been found to be clinically safe and to decrease the overall metabolic activity of hematopoietic stem cells (HSCs) and muscle [26]. However, the metabolic, functional, and molecular effects of NR treatment on ASCs and its potential to restore a more youthful function remain unresolved questions of critical importance.

In this manuscript, we aim to explore the effect of NR, on the differentiation potential and stemness characteristics of ASCs. By elucidating the underlying mechanisms involved, we hope to contribute to the development of novel strategies for regenerative medicine and adipose tissue engineering, ultimately enhancing the prospects for therapeutic applications in the context of aging and metabolic diseases.

## 2. Materials and Methods

### 2.1. Donor Specifications

Adipose tissue was collected from five non-diabetic female donors in the age range 39 ± 13 and BMI range 27 ± 4 undergoing elective plastic surgery procedures at the University of Pittsburgh Medical Center (UPMC). The procedure of tissue collection was approved by the University of Pittsburgh Institutional Review Board (IRB No. 0511186).

### 2.2. Isolation and Cultivation of Human ASCs

The isolation of adipose precursor cells was performed as follows [21,27]. Adipose tissue biopsies obtained after surgical procedures were aseptically transferred to the laboratory in a sterile sealed container. The tissue was rinsed with Dulbecco’s phosphate-buffered saline (PBS; Sigma-Aldrich, St. Louis, MO, USA), and any fibrous material and blood vessels were carefully removed through dissection. Subsequently, the tissue was cut into small pieces (approximately 1–2 mg) and was subjected to digestion in a buffer solution (Hank’s Balanced Salt Solution (HBSS) containing 200 U/mL collagenase (CLS Type II, Worthington Biochemical Corp., Lakewood, NJ, USA) and 2% *w*/*v* BSA (Sigma-Aldrich, St. Louis, MO, USA)) under continuous stirring for 60 min at 37 °C, using a ratio of 1 mg adipose tissue to 3 mL digestion buffer. Following digestion, the dispersed tissue was centrifuged at room temperature for 10 min at 200× *g*. The floating adipocytes were aspirated, and the remaining pelleted cells representing the stromal vascular fraction (SVF) were suspended in an erythrocyte lysis buffer (0.155 M NH_4_Cl, 5.7 mM K_2_HPO_4_, 0.1 mM EDTA, pH 7.3) and incubated for 10 min at room temperature. To eliminate tissue debris, the cell suspension was passed through a nylon mesh filter (100 μM pore size; Thermo Fisher Scientific, Waltham, MA, USA). The pelleted SVF obtained after another centrifugation step (10 min at 200× *g*) was resuspended in ASC medium (a mixture of Dulbecco’s Modified Eagle’s Medium (DMEM) and F-12 medium (1:1) with HEPES and L-glutamine; Thermo Fisher Scientific, Waltham, MA, USA), supplemented with 33 μM biotin, 17 μM pantothenate (both from Sigma-Aldrich, St. Louis, MO, USA), 10 ng/mL epidermal growth factor (EGF), 1 ng/mL basic fibroblast growth factor (bFGF), 500 ng/mL insulin, 2.5% fetal bovine serum, and 12.5 μM/mL gentamicin (all from Sigma-Aldrich, St. Louis, MO, USA). The SVF cells were seeded in T175 flasks at a density of 50,000 cells/cm^2^.

Once the cells reached 70% confluence, they were washed with PBS and detached using 0.05% trypsin-EDTA 1x solution (Sigma-Aldrich, St. Louis, MO, USA). The trypsin was inactivated by adding ASC medium supplemented with 10% FBS, and the cells were pelleted by centrifugation at 300× *g* for 5 min. Subsequently, the cells were seeded at a density of 5000 cells/cm^2^ and allowed to reach 70% confluence before further passaging. For this study, adipose-derived stem cells (ASCs) at passages 3–4 were used. In the experimental design, the cells were treated with various concentrations of nicotinamide riboside (Medkoo Biosciences, Morrisville, NC, USA. cat#329479).

### 2.3. Adipogenic Differentiation

To initiate adipogenesis, ASCs were seeded at a density of 50,000 cells/cm^2^ in 6-well cell culture plates and were allowed to reach confluence. The induction of adipogenesis was carried out using a differentiation medium consisting of 0.2 μM insulin, 0.5 mM 1-methyl-3-isobutylxanthine (IBMX), 0.25 μM dexamethasone, and 10 μg/mL transferrin, all obtained from Sigma-Aldrich, St. Louis, MO, USA. This differentiation medium was prepared by supplementing ASC medium. After 3 days of differentiation, the medium was replaced, and the cells were cultured in differentiation medium without IBMX for a duration of 14 days.

### 2.4. Oil Red O Staining

To visualize lipid droplets, the cells were fixed with a solution of 4% paraformaldehyde in PBS for 1 h. Subsequently, they were stained with 0.3% Oil Red O (Sigma-Aldrich, St. Louis, MO, USA) dissolved in a mixture of isopropanol and water (60:40) for 1 h. After staining, the cells were washed twice with distilled water to remove any excess stain.

### 2.5. Bodipy Staining

At day 14, following the initiation of adipogenesis, the cells were subjected to staining using Bodipy (Invitrogen, Waltham, MA, USA) and Hoechst (Sigma-Aldrich, St. Louis, MO, USA). The staining process involved incubating the cells with Bodipy and Hoechst dyes at a temperature of 37 °C for 30 min. Subsequently, images of the stained cells were captured using a fluorescent microscope. For flow cytometric analyses cells were stained with Bodipy, trypsanized, and subjected to flow cytometry analyses. Fluorescent signal was measured using a flow cytometer (Fortessa, BD, Franklin Lakes, NJ, USA), and data were analyzed using FlowJo software (BD, Franklin Lakes, NJ, USA).

### 2.6. Reactive Oxygen Species Detection

ROS levels were assessed using the fluorescent dye 2′,7′-dichlorofluorescin diacetate (DCFH2-DA) probe obtained from Invitrogen, Waltham, MA, USA. ASCs were seeded at a density of 10,000 cells per well in a 6-well plate and incubated with NR (2 mM) at 37 °C with 5% CO_2_. Following the incubation period, the ASCs were trypsinized and stained with 50 μM DCFH2-DA dye for 30 min at 37 °C. The stained cells were then analyzed using a fluorescence microscope to examine and quantify the fluorescent signal indicative of ROS levels.

### 2.7. TMRM Staining

ASCs (10,000 cells/well) were cultured in a 6-well plate, treated with different doses of NR (2 mM), and incubated at 37 °C with 100 nM TMRM (Sigma-Aldrich, St. Louis, MO, USA) for 30 min at 37 °C. Stained cells were analyzed by flow cytometry.

### 2.8. Quantitative RT-PCR Gene Expression

Total RNA was extracted using the RNeasy Micro Kit (Qiagen, Hilden, Germany), and cDNA synthesis was conducted using the RevertAid First Strand cDNA Synthesis Kit (Thermo Fisher Scientific, Waltham, MA, USA). Gene-specific primers were acquired from Sigma-Aldrich, St. Louis, MO, USA. For quantitative expression analysis, Quantstudio 3 (Thermo Fisher Scientific, Waltham, MA, USA) was employed. To normalize the mRNA quantification, β-actin was used as the reference gene. The data for each gene transcript were normalized by calculating the difference (∆Ct) between the Ct values of the housekeeping gene and the target gene. The relative increase or decrease in expression was determined by comparing the reference gene to the target gene using the ∆∆Ct method. The relative expression was calculated using the formula (=2∆∆Ct).

### 2.9. Western Blot

Western blot analysis was conducted following a similar procedure as described previously [21]. The protein levels were normalized using the “Pierce BCA Protein Assay Kit” from Thermo Fisher Scientific, Waltham, MA, USA. Cell lysates containing 15 μg of total protein per lane were prepared using a sodium dodecyl sulfate (SDS) sample buffer. Subsequently, the samples were separated by SDS-polyacrylamide gel electrophoresis (PAGE) and transferred onto polyvinylidene difluoride membranes. The membranes were then probed with the following antibodies: mouse anti-human β-actin (Proteintech, IL, USA), perilipin (Cell Signaling, MA, USA), and rabbit anti-rat IgG HRP (Proteintech, IL, USA). Densitometric analyses of the blots were performed using ImageJ software.

### 2.10. Statistical Analysis

Statistical analyses were performed in GraphPad Prism (GraphPad Software Inc., La Jolla, CA, USA). FlowJo was used for FACS analysis. The significance of the difference between means was assessed by the Student’s *t*-test or analysis of variance. Error bars are represented as the mean ± SEM. Values were significant at *p* values of <0.05 (* *p* < 0.01 and # *p* < 0.05).

## 3. Results

### 3.1. Adipocyte Differentiation of Human Adipose-Derived Stem Cells Is Associated with Increased Mitochondrial Activity and ROS Production

**Figure 1:**
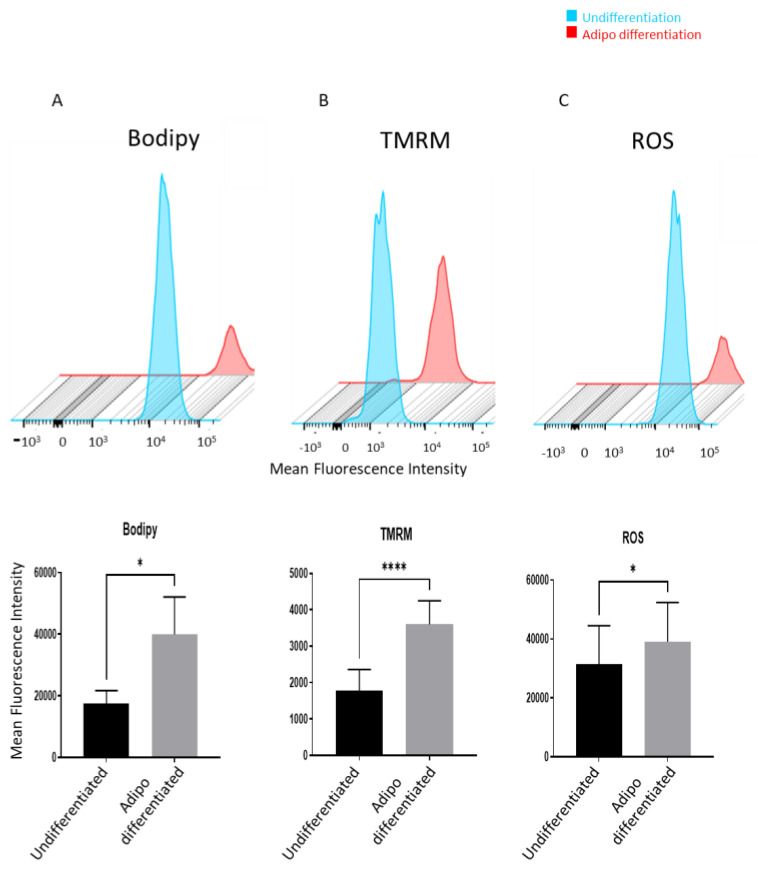
(**A**) Mean fluorescence intensity (MFI) of differentiated and undifferentiated hASCs stained with Bodipy stain was measured by flow cytometry at day 14 post-adipogenesis (n = 3). (**B**) Mean fluorescence intensity of differentiated and undifferentiated hASCs stained with TMRM stain was measured by flow cytometry at day 14 post-adipogenesis (n = 3). (**C**) Differentiated and undifferentiated ASCs were stained with DCFDA dye and mean fluorescence intensity (MFI) was measured by employing flow cytometry (n = 3). * *p* < 0.05, **** *p* < 0.00005.

We induced adipocyte differentiation in cultured expanded adipose-derived stem cells (ASCs) for a duration of 14 days. To confirm successful differentiation, we measured the accumulation of Bodipy dye using flow cytometry. The results demonstrated a significant increase in the mean fluorescence intensity of Bodipy in the differentiated cells, indicating successful adipocyte differentiation (Figure 1A). Maintaining lower mitochondrial activity is crucial for undifferentiated progenitor cells to preserve their stemness and undifferentiated state [28,29,30]. To compare the mitochondrial activity between undifferentiated and adipocyte-differentiated ASCs, we measured the uptake and retention of TMRM (tetramethylrhodamine methyl ester) using flow cytometry, a read out of mitochondrial activity via measuring mitochondria membrane potential. Adipocyte-differentiated cells exhibited a significantly higher level of TMRM accumulation, as reflected by the increased fluorescence intensity (Figure 1B). In addition, we analyzed the levels of reactive oxygen species (ROS) in both undifferentiated and differentiated adipocytes. Consistent with the observed increase in mitochondrial activity, differentiated ASCs displayed significantly higher levels of ROS compared to undifferentiated cells (Figure 1C). These data suggest that adipocyte differentiation is associated with elevated mitochondrial activity and increased ROS levels. These findings highlight the metabolic changes that occur during adipocyte differentiation of ASCs. The higher mitochondrial activity and ROS levels observed in differentiated adipocytes signify the dynamic cellular processes involved in adipogenesis.

### 3.2. Nicotinamide Riboside Treatment Reduces Terminal Adipocyte Differentiation of hASCs

**Figure 2:**
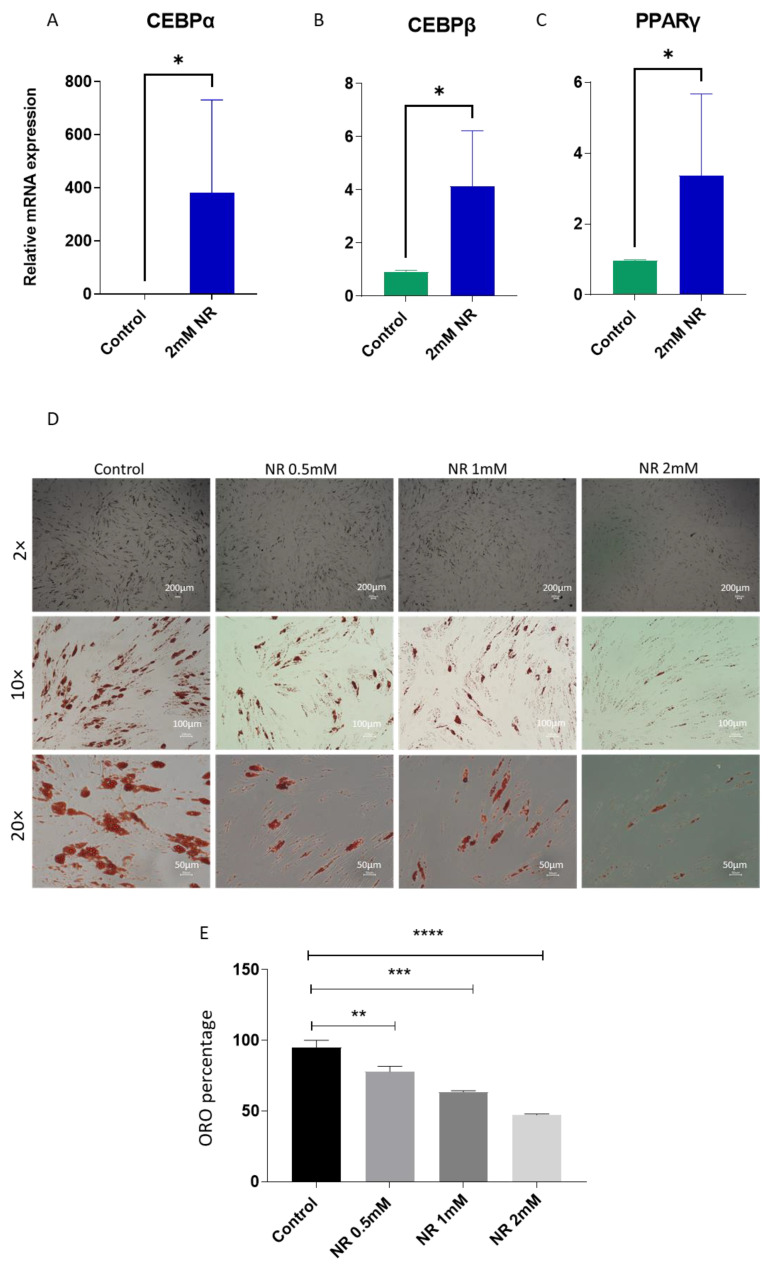
(**A**–**C**) hASCs were treated with control media or 2 mM NR containing media for 7 days. Cells were lysed for RNA extraction and reverse-transcribed for real-time PCR quantification of CEBPα (**A**), CEBPβ (**B**), and PPARγ (**C**) genes (n = 3). (**D**) hASCs were grown to confluence in 6-well plates and differentiated in the absence or presence of different concentrations of NR. On day 14 post-differentiation, cells were fixed and stained with Oil-Red-O stain and imaged at different magnifications, as indicated. (**E**) Percentage Oil-Red-O positive cells were counted and plotted. * *p* value < 0.05, ** *p* < 0.005, *** *p* < 0.0005, **** *p* < 0.00005.

Previous studies have presented conflicting findings regarding the role of nicotinamide mononucleotide in regulating adipogenesis in mesenchymal stem cells derived from various tissue sources [31,32]. In this study, we focused on human adipose-derived stem cells (hASCs) and investigated the effects of nicotinamide riboside (NR), a readily cell permeable NAD^+^ precursor on adipogenesis. To assess the impact of NR on adipogenesis, we cultured hASCs for 7 days with NR and examined the expression of key transcriptional factors involved in adipocyte differentiation, namely CEBPα, CEBPβ, and PPARγ. These factors play a crucial role in regulating the process of adipogenesis. Remarkably, our analysis revealed a significant upregulation of all three transcriptional factors following NR treatment (Figure 2A–C). These findings suggest a potential positive role of NR in supporting adipogenesis. To further evaluate the effect of NR on terminal adipogenesis and lipid accumulation, we treated hASCs with varying concentrations (0.5, 1, and 2 mM) of NR for 7 days before initiating adipogenesis. Subsequently, we continued NR treatment during the 14-day differentiation period. Surprisingly, when we stained the differentiated cells with Oil-Red-O, a marker for lipid accumulation, we observed a dose-dependent decrease in the number of Oil-Red-O positive cells (Figure 2D,E).

**Figure 3:**
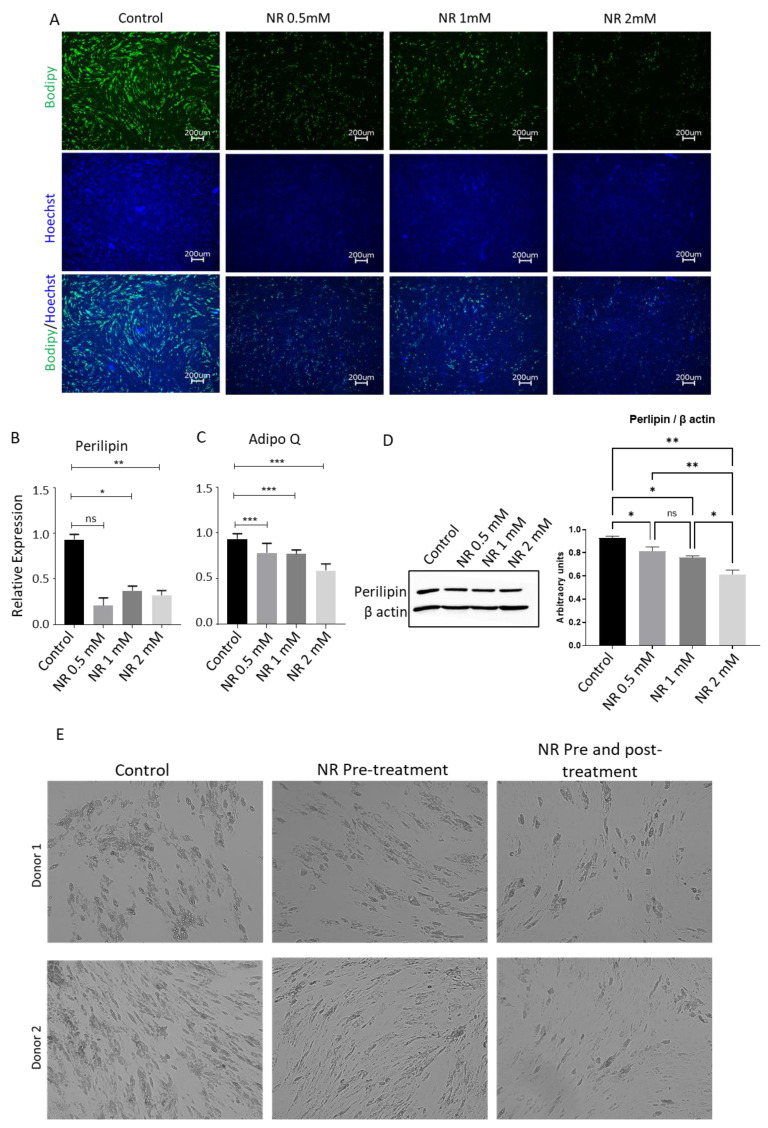
(**A**) hASCs were grown to confluence in 6-well plates and were differentiated in the absence or presence of different concentrations of NR. On day 14 post-differentiation induced cells were fixed and stained with Bodipy (Green) and Hoechst (Blue) and imaged using fluorescence microscope. (**B**,**C**) hASCs were differentiated in the presence or absence of different concentrations of NR. On day 14 post-differentiation induction cells were lysed for RNA extraction and reverse-transcribed for real-time PCR quantification of Perilipin (**B**), and AdipoQ (**C**) genes. (**D**) hASCs were differentiated in the presence or absence of different concentrations of NR. On day 14 post-differentiation induction cells were lysed for protein extraction and analyzed for Perilipin protein by western blotting. Perilipin protein western blot band intensity was analyzed by Image J and normalized to β actin housekeeping control. (**E**) hASCs were treated with 2 mM NR. NR was washed from the pre-treatment group and differentiation was induced in the absence of NR. In the NR pre- and post-treatment group hASCs were treated for 7 days with 2 mM NR and NR was also added to differentiation media for 14 days. Cells were imaged at day 14 post-differentiation. Control wells were never treated with NR. * *p* < 0.01, ** *p* < 0.001, *** *p* < 0.0001.

To validate this observation, we performed Bodipy staining, another lipid marker, on differentiated ASCs, which consistently demonstrated a similar trend (Figure 3A).

To gain further insights into the effect of NR on adipogenesis at the molecular level, we conducted quantitative real-time polymerase chain reaction (qRT-PCR) analyses of two differentiation-related genes: *Perilipin1* and *Adipo Q* [21]. Perlipin 1 is a late differentiation marker for adipocytes and coats the lipid droplet, while AdipoQ (adiponectin) is the protein hormone secreted highly specifically by mature adipocytes. Our results demonstrated a significant reduction in the expression of these genes upon NR treatment (Figure 3B,C). Additionally, we confirmed the decrease in *Perilipin* gene expression at the protein level using western blotting, which indicated lower levels of the adipocyte differentiation marker protein at day 14 post-differentiation (Figure 3D). These terminal differentiation results were contradictory to the initial upregulation of the key transcriptional factors essential for adipocyte differentiation, suggesting that despite the increased expression of adipogenesis-supporting transcription factors, NR treatment impedes the terminal differentiation of adipocytes.

### 3.3. NR Induces Hallmark Stemness Gene Expression in hASCs and Reduces Mitochondrial Activity

**Figure 4:**
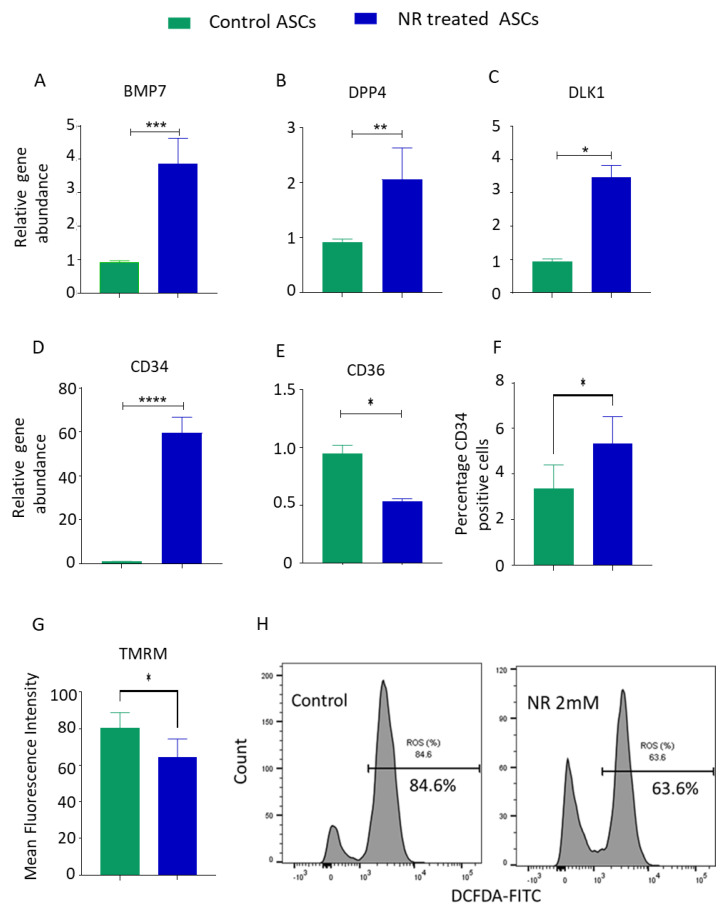
(**A**–**E**) hASCs were treated with control media or 2 mM NR for 7 days. Cells were lysed for RNA extraction and reverse-transcribed for real-time PCR quantification of *BMP7* (**A**), *DPP4* (**B**), *DLK1* (**C**), *CD34* (**D**), and *CD36* (**E**) genes. (**F**) hASCs were treated with NR or control and analyzed by flow cytometry for the surface expression of *CD34*. (**G**) Control or NR-treated ASCs were stained with TMRM and analyzed by flow cytometry. (**H**) NR-treated ASCs were stained with DCFDA dye and analyzed by flow cytometry. * *p* value < 0.05, ** *p* < 0.005, *** *p* < 0.0005, **** *p* < 0.00005.

To gain a deeper understanding of the role of NR treatment in blocking terminal adipocyte differentiation, we conducted experiments to compare the effects of NR treatment during the differentiation phase versus pre-differentiation treatment alone. Human adipose-derived stem cells (hASCs) were treated with NR for 7 days and then differentiated in NR-free media for an additional 14 days. In the comparative group, hASCs were pretreated and differentiated in the presence of NR. Surprisingly, both groups exhibited lower adipocyte differentiation (Figure 3E), indicating that NR treatment prior to the induction of differentiation was sufficient to block terminal adipocyte differentiation. Previous studies have suggested that hASCs with higher expression of stemness genes exhibit resistance to adipogenesis induction and maintain an undifferentiated status [28,33]. To assess whether NR treatment enhances the stemness characteristics of hASCs, we examined the expression of hallmark stemness genes in NR-treated hASCs. The results demonstrated a significant increase in the expression of BMP7, *DPP4*, *DLK1*, and *CD34* (Figure 4A–D), which are genes associated with maintaining the stemness of adipose progenitor cells. Interestingly, NR treatment led to the downregulation of CD36, a gene associated with adipocyte differentiation (Figure 4E). CD34 surface expression is known to be lost during cell culture expansion in hASCs research. Remarkably, we observed that NR treatment resulted in an increase in the surface expression of *CD34* (Figure 4F). Furthermore, lower mitochondrial activity has been associated with the maintenance of stemness. TMRM staining of NR-treated hASCs revealed a significant decrease in mitochondrial membrane potential (Figure 4G). Additionally, NR-treated hASCs exhibited lower production of reactive oxygen species (ROS) (Figure 4H). In summary, our findings suggest that NR-mediated reduction in mitochondrial activity contributes to the maintenance of stemness in hASCs and enables them to resist adipogenic differentiation pressure. The upregulation of stemness genes, such as *BMP7*, *DPP4*, *DLK1*, and *CD34*, along with the downregulation of the adipocyte differentiation gene CD36, further supports the notion that NR treatment enhances the stemness characteristics of hASCs. These insights provide valuable information about the mechanisms underlying NR’s effects on hASCs and their potential implications for adipose tissue biology and regenerative medicine.

## 4. Discussion

Adipose stem cells (ASCs) play a pivotal role in the regeneration of adipose tissue by engaging in self-renewal and differentiation processes, which contribute to the maintenance of the tissue’s stem cell pool. ASCs possess the remarkable ability to proliferate and differentiate into adipocytes, allowing them to replace damaged or depleted adipose tissue. However, the aging process and obesity have been found to negatively impact the stemness properties of ASCs [28].

As individuals age, there is a gradual decline in the regenerative capacity of ASCs. This decline is characterized by a reduction in their ability to self-renew and differentiate into adipocytes. The loss of stemness properties in aged ASCs is often associated with decreased proliferation potential, altered gene expression profiles, and impaired differentiation capabilities [15]. These age-related changes contribute to the overall decline in adipose tissue function and may contribute to metabolic disorders associated with aging [17]. Similarly, obesity has been linked to the alteration of ASCs’ stemness properties. Excessive accumulation of adipose tissue due to obesity disrupts the normal cellular microenvironment and leads to adipose tissue dysfunction. Excessive calories in the form of fat and carbohydrates result in adipose tissue expansion via an increase in adipocyte number (hyperplasia) and size (hypertrophy) [34]. A major mechanism of size expansion is the uptake of lipids through fatty acid scavenger, CD36, and the conversion of glucose to carbohydrates through lipogenesis [35,36]. Long-term overnutrition results in fragile adipocytes and exhausted senescent ASCs that lead to adipose tissue and systemic inflammation and consequently metabolic disorder [36,37,38]. In this context, ASCs undergo phenotypic and functional changes that compromise their ability to effectively regenerate adipose tissue [8]. The process of differentiating ASCs into adipocytes is accompanied by increased energy demands and metabolic rates, leading to elevated mitochondrial activity and the generation of reactive oxygen species (ROS). Higher levels of ROS can drive stem cells towards differentiation, leading to senescence and cell death [39]. In our study, using hASCs as an adipocyte differentiation model, we also observed an increase in the mitochondrial activity and ROS production upon differentiation of ASCs to adipocytes.

The exact mechanism of how oxidative stress inhibits adipocyte differentiation is not known, but most of the studies link impaired adipogenesis to mitochondrial dysfunction [17,40]. Chronic inflammation and oxidative stress result in a reduction in NAD^+^ biosynthesis and mitochondrial levels, leading to dysfunction and ROS production. Increased ROS levels further result in the downregulation of mitochondrial complex I and II [17,41]. In addition, oxidative stress inhibits mitotic expansion, a critical step involved in adipocyte differentiation via the repression of E2F target genes cyclin A and MCM7, most likely through an epigenetic modification [17].

In the past, the aging process has been thought to result in an irreversible decline in the functionality of stem cells. Stem cells are crucial for tissue regeneration and repair, but as organisms age, their regenerative capacity diminishes. However, recent scientific advancements have challenged this conventional understanding and shed light on the importance of maintaining nicotinamide adenine dinucleotide (NAD) homeostasis. NAD is a coenzyme involved in numerous cellular processes, including energy metabolism, DNA repair, and the regulation of gene expression. It plays a fundamental role in cellular functions and has been found to decline with age [42]. This decline in NAD levels is linked to age-related impairments in tissue-resident stem cells. Recent studies have revealed that replenishing NAD levels can have a significant impact on the functionality of stem cells, offering potential therapeutic benefits [23,24,25,26]. By restoring NAD homeostasis, it is possible to counteract the age-related functional decline observed in various tissue-resident stem cells. Replenishing NAD levels using NR has shown promising results in improving the regenerative potential of stem cells in different tissues, including skeletal muscle, brain, and skin [23,26]. The current data reveal that NR treatment reduces terminal adipocyte differentiation of ASCs. Although we observed a higher transcription of key adipogeneic transcription factors, the terminal outcome is lower adipogenesis. Our study highlighted this interesting mechanistic regulation in the adipogeneic transcriptional players in the presence of NR. A previous study using NAD showed a reduction in terminal differentiation in hASCs [14]. Although NAD treatment showed lower PPARγ expression at day 8 the levels were significantly higher at an earlier time point at day 4 [14]. NAD-mediated SIRT1 regulation contributed to terminal differentiation blockade [14]. Our results demonstrated that a pre-treatment of hASCs with NR is sufficient to reduce the differentiation cascade.

**Figure 5:**
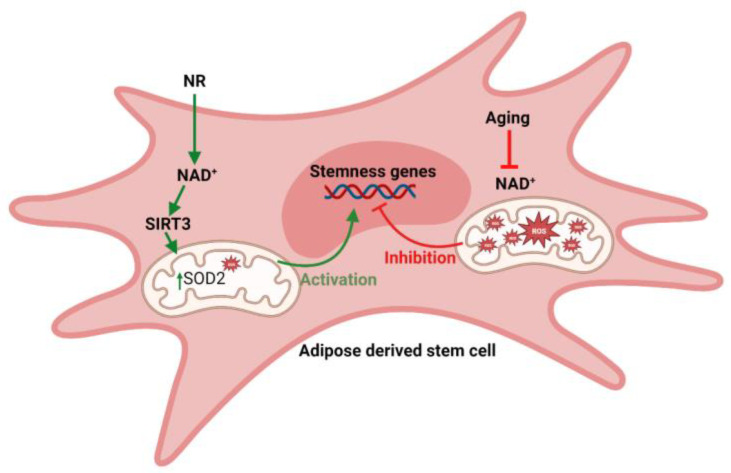
Possible mechanism of action of NR on ASCs stemness.

The mechanism behind NAD’s rejuvenating effects on stem cells is multifaceted. NAD is crucial for maintaining the balance between cellular energy production and consumption. It activates enzymes such as sirtuins, which are involved in regulating cellular metabolism, stress response, and longevity. Additionally, NAD is involved in DNA repair processes, which play a critical role in preserving the genomic integrity of stem cells. By replenishing NAD levels, it is possible to enhance the metabolic and functional properties of stem cells. This can lead to improved cellular energy production, increased DNA repair capacity, and enhanced cellular resilience to stressors. As a result, stem cells regain their regenerative potential and can contribute more effectively to tissue repair and rejuvenation. Our study showed, for the first time, the increase in the stemness signature of hASCs by NR treatment. We observed an increase in the expression of *BMP7*, *DPP4*, *DLK1*, and *CD34*. NR treatment also resulted in an increase in the surface expression of *CD34* on hASCs. Studies have shown that adipocyte progenitor cells having higher stemness features resist differentiation pressure [33]. It is most likely that the NR-mediated increase in stemness for hASCs contributed to the resistance to differentiation. Our results demonstrated that NR treatment results in a decrease in mitochondrial activity reflected by TMRM staining and ROS activity. It is most likely that NR treatment results in increased NAD^+^ levels that drive the ROS production via a SIRT3-dependent deacetylation and the stimulation of superoxide dismutase (*SOD2*) (Figure 5) [43,44]. The exact mechanism of how NR enhances the stemness features in hASCs needs further investigation.

The emerging field of NAD therapeutics offers a promising avenue for combating age-related decline in stem cell functionality. By targeting NAD homeostasis, researchers aim to develop interventions that can restore the youthful characteristics of stem cells and enhance their regenerative abilities. The failure of adipose tissue regeneration with age is a key contributor to the development of age-associated metabolic disorders. Further studies and clinical trials are necessary to explore the full potential of NAD-based therapies in combating age-related adipose tissue degeneration and promoting healthier aging.

## Data Availability

The data presented in this study are available on request from the corresponding author.
